# Evaluating the Applicability of Existing Lexicon-Based Sentiment Analysis Techniques on Family Medicine Resident Feedback Field Notes: Retrospective Cohort Study

**DOI:** 10.2196/41953

**Published:** 2023-07-27

**Authors:** Kevin Jia Qi Lu, Christopher Meaney, Elaine Guo, Fok-Han Leung

**Affiliations:** 1 Department of Family and Community Medicine University of Toronto Toronto, ON Canada; 2 Department of Economics University of Toronto Toronto, ON Canada

**Keywords:** medical education, medical resident, feedback, field note, text mining, data mining, sentiment analysis, lexicon, lexical, dictionary, dictionaries, vocabulary, resident, medical student, medical trainee, residency, utility, feasibility

## Abstract

**Background:**

Field notes, a form for resident-preceptor clinical encounter feedback, are widely adopted across Canadian medical residency training programs for documenting residents’ performance. This process generates a sizeable cumulative collection of feedback text, which is difficult for medical education faculty to navigate. As sentiment analysis is a subfield of text mining that can efficiently synthesize the polarity of a text collection, sentiment analysis may serve as an innovative solution.

**Objective:**

This study aimed to examine the feasibility and utility of sentiment analysis using 3 popular sentiment lexicons on medical resident field notes.

**Methods:**

We used a retrospective cohort design, curating text data from University of Toronto medical resident field notes gathered over 2 years (from July 2019 to June 2021). Lexicon-based sentiment analysis was applied using 3 standardized dictionaries, modified by removing ambiguous words as determined by a medical subject matter expert. Our modified lexicons assigned words from the text data a sentiment score, and we aggregated the word-level scores to a document-level polarity score. Agreement between dictionaries was assessed, and the document-level polarity was correlated with the overall preceptor rating of the clinical encounter under assessment.

**Results:**

Across the 3 original dictionaries, approximately a third of labeled words in our field note corpus were deemed ambiguous and were removed to create modified dictionaries. Across the 3 modified dictionaries, the mean sentiment for the “Strengths” section of the field notes was mildly positive, while it was slightly less positive in the “Areas of Improvement” section. We observed reasonable agreement between dictionaries for sentiment scores in both field note sections. Overall, the proportion of positively labeled documents increased with the overall preceptor rating, and the proportion of negatively labeled documents decreased with the overall preceptor rating.

**Conclusions:**

Applying sentiment analysis to systematically analyze field notes is feasible. However, the applicability of existing lexicons is limited in the medical setting, even after the removal of ambiguous words. Limited applicability warrants the need to generate new dictionaries specific to the medical education context. Additionally, aspect-based sentiment analysis may be applied to navigate the more nuanced structure of texts when identifying sentiments. Ultimately, this will allow for more robust inferences to discover opportunities for improving resident teaching curriculums.

## Introduction

Competency-based medical education emphasizes skills development and educational outcome measures (eg, entrustable professional activities) designed within an individualized timeline of progression [1]. One increasingly adopted tool used in competency-based medical education across Canadian medical training programs is field notes. Preceptors fill out these structured feedback forms for residents, evaluating their “Strengths” and “Areas of Improvement” in a clinical encounter. They are a qualitative way to track learner progress and improve feedback documentation [2]. Residents believe that using field notes increases feedback volume [3], focuses the feedback, and makes the feedback more useful overall [4].

Methods that computationally summarize the growing amounts of text data from field notes are needed. In their raw form, extensive text collections from field notes are difficult for faculty program leaders to navigate. Efficient strategies to synthesize and compare the sentiment in field notes are valuable for evaluating information to help improve the teaching curriculum.

Sentiment analysis is a subfield of text mining or natural language processing [5]. It is the process of computationally detecting whether a piece of text is inherently positive, neutral, or negative. In health care, sentiment analysis has been used to monitor public health care concerns on social media [6] and to synthesize patient reviews of health care services in England [7]. Despite rising interest in machine learning tools, sentiment analysis has been applied sparingly to medical education and resident performance evaluation [8].

In this study, we assess the feasibility and utility of using sentiment analysis to synthesize a large corpus of medical education field notes. We apply 3 commonly employed sentiment lexicons (ie, BING, AFINN, and NRC) [9]. In health care, the 3 lexicons have been comparatively evaluated on tweets from nurses during the COVID-19 pandemic [10] and electronic health records for suicide risk assessments [11]. We will be the first to use these lexicons to analyze feedback generated in resident-preceptor field note performance evaluations. Quantitatively summarizing sentiment information from field notes will allow for subsequent analysis that may reveal valuable insights for medical education program design. For example, predicted sentiment scores can be correlated with learning parameters such as teaching locations or type of patient encounter. Predicted sentiment scores can also be correlated with resident and preceptor characteristics to preemptively identify residents falling behind and preceptors who might apply systematically different evaluation standards from others. All these results inform essential decision-making regarding improving a training program.

## Methods

### Study Design and Setting

The study used a retrospective cohort design. We used clinical encounter–based field notes written between July 1, 2019, and June 30, 2021, by preceptors for family medicine residents from 14 training sites affiliated with the University of Toronto’s Department of Family and Community Medicine.

In field notes, preceptors write comments on their perception of the strengths and areas of improvement of the resident’s performance during a clinical encounter. Preceptors also provide an overall performance rating for the clinical encounter on a 5-point Likert scale, with 1 indicating the poorest and 5 indicating the best performance. The categories that preceptors fill out in the field note template used at the University of Toronto’s Department of Family and Community Medicine are as follows: assessee, date of encounter, state of residency, assessment tool (CanMEDs roles), rotation service, site, area(s) of observation, level of performance or competency (5 levels), strengths, and actions (areas of improvement).

### Sentiment Lexicons

We applied lexicon-based sentiment analysis using 3 well-established sentiment dictionaries: BING, AFINN, and NRC. The BING dictionary was first designed around the domain of e-commerce customer reviews [12]; AFINN was created for synthesizing Twitter microblogs [13]; and NRC was a large, crowdsourced lexicon geared toward a more generalized domain [14]. We reported the number of unique words in each lexicon and the number of unique words labeled by each lexicon within our text data. From this subset, a single subject matter expert (KL) then labeled and removed words deemed ambiguous in the context of medical resident clinical teaching; another study team member (CM) reviewed and adjudicated decisions regarding ambiguous words identified by KL.

### Statistical Analysis

Text was extracted from preceptor-resident field notes from the “Strengths” and “Areas of Improvement” sections, and word-level sentiment analysis was applied to these sections, respectively. On the word level, we identified the most prevalent words of each sentiment in each section and calculated their frequencies. On a document level, a sentiment score output was generated by computing the mean polarity of all words labeled. Documents were further classified as positive, neutral, or negative based on their sentiment score.

Agreement between the 3 sentiment dictionaries was evaluated by calculating Cohen weighted kappa statistics.

To assess the concurrent validity of the sentiment scores, we measured the association between our derived document-level sentiment scores and overall preceptor ratings (measured on a 5-point Likert scale).

### Ethics Approval

Approval for this study was obtained from the University of Toronto research ethics board (protocol 41745).

## Results

### Overview

Between July 1, 2019, and June 30, 2021, a total of 20,455 field notes written across 14 resident training sites affiliated with the University of Toronto Department of Family and Community Medicine were included in the analysis. Of them, 20,452 field notes contained a “Strengths” text entry, and 20,411 field notes had an “Areas for Improvement” entry. The median number of words for the strengths text was 28 (IQR 16-44), whereas the median length of the areas for improvement text was 14 (IQR 4-29) words. The study sample included 662 unique residents and 500 unique preceptors. The median number of field notes per resident was 27 (IQR 13-44), whereas the median number of field notes per preceptor was 22 (IQR 5-59). Completion of a field note was not mandatory after clinical encounters.

### Word-Level Sentiment Analysis: Restricted Applicability of Established Sentiment Lexicons in Field Note Feedback

The following 3 lexicon dictionaries were individually used to assess the sentiment of field note text: AFINN, BING, and NRC. The degree of applicability was assessed for each dictionary by evaluating the proportion of ambiguous words out of the total number of unique words labeled by our corpus (Table 1).

**Table 1:** Proportion of ambiguous words labeled in field note text data by three standard lexicon dictionaries.

Dictionary	Unique words in dictionary, n	Unique words labelled, n	Ambiguous words labelled, n	Proportion of ambiguity
				
AFINN	2477	1081	305	0.282
BING	6780	1885	550	0.291
NRC	5464	2039	720	0.353

The 3 dictionaries showed a similarly restricted level of applicability when applied to our medical education field note corpus. About a third of all uniquely labeled words across all 3 dictionaries were labeled as ambiguous, with the NRC lexicon having a slightly higher ambiguity rate than the others.

Ambiguous words also tended to appear with high frequencies. Table S1 (Multimedia Appendix 1) lists the 5 most frequent sentiment-labeled words in each of the 3 dictionaries; the majority are ambiguous. We removed these ambiguous words from the original dictionaries to create modified dictionaries, which improved applicability in our research domain. Based on the modified dictionaries, the top 5 words were mainly those expressing affirmative and critical sentiments. However, mechanically, the modified dictionaries had poorer coverage and labeled fewer words in our text. For example, the 5 most frequent negative sentiment-labeled words by the “unmodified” AFINN dictionary in the “Strengths” section of field notes cover 4597 occurrences. In contrast, those labeled by the “modified” dictionary only cover 519 occurrences, an 88.7% decrease.

### Document-Level Sentiment Analysis

#### Overall Field Note Sentiment Scores

The mean sentiment score output for the “Strengths” and “Areas of Improvement” sections for all field notes for each of the 3 lexicon dictionaries were computed. Increasing positive values indicate greater positive sentiment. Decreasing negative values indicate increased negative sentiment. Across all 3 lexicons, the average sentiment for the “Strengths” section was determined to be very mildly positive (AFINN: average of 0.12988 on a scale of –5 to 5; BING: average of 0.06619 on a scale of –1 to 1; and NRC: average of 0.08382 on a scale of –1 to 1). Compared to the “Strengths” section, the mean sentiment across all 3 lexicons for the “Areas of Improvement” section was also very mildly positive, but it was less positive than that of the “Strengths” section (0.05654 for AFINN, 0.02839 for BING, and 0.06014 for NRC).

#### Agreement Level Between the Lexicons for Discrete Sentiment Labels Across Individual Field Notes

There was reasonable agreement between the modified dictionaries with respect to document-level sentiment classification for the “Strengths” text as shown via the weighted kappa estimates (AFINN vs BING: 0.61, 95% CI 0.60-0.62; AFINN vs NRC: 0.48, 95% CI 0.45-0.51; and BING vs NRC: 0.45, 95% CI 0.42-0.48).

Comparably, the weighted kappa estimate for unmodified dictionaries was consistently lower but still showed moderate agreement. Similar trends were observed when estimating agreement across the modified dictionaries applied to the “Areas of Improvement” section.

#### Sentiment Score Associations With Overall Preceptor Rating

**Figure 1:**
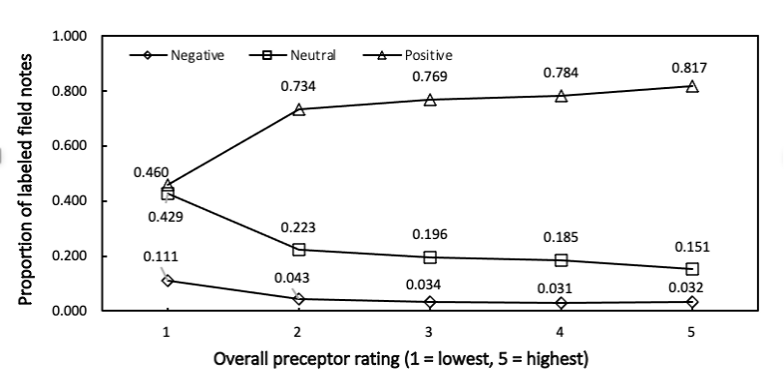
Proportions of field notes classified as sentiment negative, neutral, positive in the “Strengths” section based on the modified BING dictionary, by “clinical encounter overall rating” strata of 1 (low) to 5 (high).

**Figure 2:**
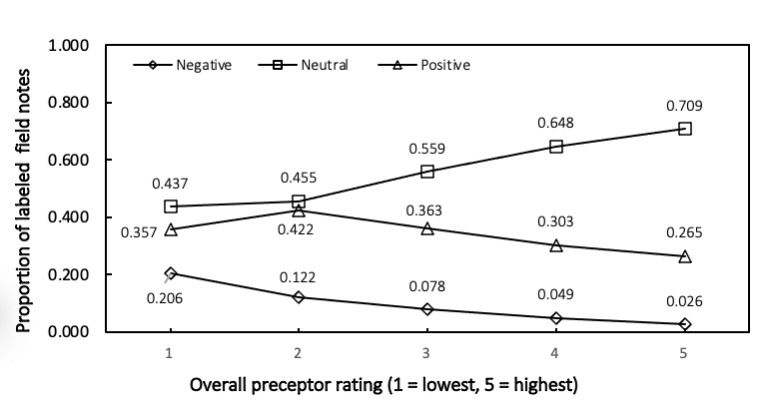
Proportions of field notes classified as sentiment negative, neutral, positive in “Areas of Improvement” section based on the modified BING dictionary, by “clinical encounter overall rating” strata of 1 (low) to 5 (high).

We examined the association between document-level sentiment classifications and overall preceptor ratings shown in Figure 1 and Figure 2.

In the “Strengths” section of field notes, a higher preceptor rating was associated with a higher proportion of positively labeled field notes and a decreasing proportion of neutral and negatively labeled field notes across all 3 modified dictionaries (only BING is shown in Figure 1; AFINN and NRC are shown in Table S2 in Multimedia Appendix 1). The greatest proportion of field notes for the “Strengths” section for each preceptor rating was labeled positive, and the smallest proportion was labeled negative.

In the “Areas of Improvement” section of field notes, a higher preceptor rating was associated with a higher proportion of neutrally labeled field notes, a decreasing proportion of negatively labeled field notes, and a decreasing proportion of positively labeled field notes (except between ratings 1 and 2, where the greatest proportion of field notes was labeled neutral and the smallest proportion was labeled negative).

## Discussion

### Principal Findings

In our study, we found that it is feasible to apply sentiment analysis with 3 common lexicons to medical education field notes. The “Strengths” section had a mildly positive sentiment, and the “Areas of Improvement” section had slightly lower sentiment, as expected. We also observed that in the “Strengths” section, a higher preceptor rating was associated with a higher proportion of positively labeled field notes; and in the “Areas of Improvement” section, a lower preceptor rating was associated with a higher proportion of negatively labeled field notes, which we believe serves as concurrent validity. Using sentiment analysis, we efficiently analyzed the sentiment of over 20,000 field notes and evaluated the quality of the predictions by benchmarking our predicted sentiment scores against quantitative preceptor ratings also provided in our field notes.

Although this study was a useful first attempt at applying sentiment analysis to field notes, some challenges restricted the utility of this approach. First, high frequencies of ambiguous words appear in medical education clinical settings. An example of an ambiguous word is “patient,” which generally has a positive connotation when used as an adjective, but in a medical context, it will very often refer to the person receiving medical treatment. Similarly, the word “pain” may generally have a negative connotation, but in a medical context, it most likely describes what a patient is experiencing. We attempted to address this challenge by removing perceived ambiguous words by a subject expert. However, even after the modification, there were still many scoring inconsistencies. An inconsistent example with a negative sentiment score was the following positive feedback in the “Strengths” section: “Thorough history, complete pertinent negatives.”

### Limitations

Accordingly, the first limitation of our study is the potential for incorrect sentiment scoring when applying a lexicon to a domain for which it was not specifically constructed [15]. Potentially relevant sentimental terms in a medical context might have been excluded, and many ambiguous words were included. Removing ambiguous words improved accuracy but reduced coverage, which raises the challenge of balancing the trade-off between removing ambiguous words and having a fair representation of field note corpus through labeled words to capture its polarity and context reliably.

Another limitation is the way preceptors may write feedback. Feedback effectiveness is related to how focused the feedback is on the behaviors or actions of the trainee, with emphasis on clear learning objectives [16]. Within our field note corpus, the median feedback length was 1-2 sentences, although occasionally, it was as short as one word. This limited word count, often representing nonfocused feedback, restricted the ability to detect particular sentiments. Further, such short text is more likely to be skewed, often inaccurately, by 1-2 words with strong polarity. Western culture also emphasizes providing constructive feedback, which aims to be nonjudgmental and not overly harsh [17] and can further skew polarity toward being more positive.

Critical insight can be extracted from trends correlating learner sentiment with different learning parameters. Specific learner competencies, patient presentations, or training sites may be associated with a particular sentiment. For example, residents may receive more negative than positive feedback with certain clinical encounters. Specific preceptors may provide more positive or negative feedback. This valuable information can drive timely exploration for faculty and support decision-making, such as adjusting learner curriculums, optimizing teaching sites, or even offering feedback training. As more data are gathered, analysis can be applied to trend and compare sentiment over time, such as across cohorts. We established the feasibility of applying sentiment analysis to resident-preceptor feedback but also uncovered some limitations that can help guide further optimization.

Future studies can focus on constructing a lexicon that accurately represents the vocabulary used in a medical education clinical setting, with a goal for 90% accuracy, which is the average target for domain-specific lexicons [18]. This may be achieved by taking a sample of existing field notes and having subject experts label pertinent words based on a new discrete sentiment scale. Since a word’s sentiment depends on the context in which it is used, labeling and scoring can be adjusted to context accordingly. Alternatively, aspect-based sentiment analysis can be applied to detect sentiments within aspects of clinical encounters, such as history taking or physical exams.

### Conclusions

In the context of postgraduate family medicine education, a growing collection of text data is generated from preceptor-resident feedback field notes. Sentiment analysis can be used to analyze the appraisals entailed in these field notes efficiently and systematically. We observed that 3 established lexicons could be feasibly applied, although with limited accuracy, due to a significant proportion of ambiguous words present in the clinical context and short feedback length. Accordingly, future work should aim to generate a domain-specific dictionary for medical training and use in combination with an aspect-based sentiment analysis technique. The efficient analysis of large collections of valuable feedback text to explore trends and correlations with clinical encounter characteristics will be instrumental in improving medical training quality.

## Appendix

Multimedia Appendix 1 Table S1 and Table S2.
