# Supplementary material for: Evaluating the Applicability of Existing Lexicon-Based Sentiment Analysis Techniques on Family Medicine Resident Feedback Field Notes: Retrospective Cohort Study
Source: JMIR Med Educ. 2023 Jul 27;9:e41953. doi: 10.2196/41953 (PMC10415947; doi:10.2196/41953)
Supplement: Multimedia Appendix 1 [file mededu_v9i1e41953_app1.pdf]

Table S1. Positive and negative words with highest frequencies (freq.) across all field note “Strengths” and “Areas of Improvement” sections based on original and modified (mod.) dictionaries.

AFINN (Freq)	AFINN Mod (Freq)	BING (Freq)	BING Mod (Freq)	NRC (Freq)	NRC Mod (Freq)
**Strengths - positive words**					
Good (7769)	Good (7769)	Patient (13949)	Good (7769)	Patient (13949)	Good (7769)
Excellent (5541)	Excellent (5541)	Good (7769)	Excellent (5541)	Good (7769)	Excellent (5541)
Great (2207)	Great (2207)	Excellent (5541)	Appropriate (3681)	Excellent (5541)	Knowledge (629)
Care (2179)	Clear (709)	Appropriate (3681)	Great (2207)	Management (3333)	Comprehensive (619)
Help (814)	Comprehensive (619)	Well (3380)	Rapport (993)	Job (2036)	Understanding (452)
**Strengths – negative words**					
Pain (2107)	Vague (123)	Pain (2107)	Challenging (321)	Pain (2107)	Spent (150)
Risk (764)	Poor (101)	Symptoms (2002)	Negatives (205)	Diagnosis (1172)	Vague (123)
Anxiety (677)	Limited (99)	Issues (1661)	Limitations (159)	Chronic (882)	Stress (106)
No (643)	Pressure (99)	Concerns (1643)	Vague (123)	Case (827)	Limited (99)
Difficult (406)	Missed (97)	Complex (927)	Stress (106)	Risk (764)	Pressure (99)
**Areas of improvement – positive words**					
Good (1362)	Good (1362)	Patient (4024)	Good (1362)	Patient (4024)	Good (1362)
Help (1009)	Great (715)	Work (1505)	Great (715)	Continue (2715)	Learning (403)
Care (984)	Helpful (400)	Good (1362)	Appropriate (624)	Management (1563)	Helpful (400)
Ensure (803)	Excellent (384)	Well (1251)	Helpful (400)	Good (1362)	Excellent (384)
Great (715)	Better (296)	Great (715)	Excellent (384)	Visit (878)	Knowledge (282)
**Areas of improvement – negative words**					
No (1109)	Forget (275)	Issues (757)	Challenging (173)	Case (941)	Forget (275)
Pain (606)	Avoid (196)	Symptoms (704)	Hard (109)	Diagnosis (696)	Avoid (196)
Risk (438)	Hard (109)	Concerns (642)	Missed (89)	Pain (606)	Pressure (104)
Forget (275)	Pressure (104)	Pain (606)	Stress (81)	Risk (438)	Small (104)
Difficult (239)	Missed (89)	Risk (438)	Limited (69)	Forget (275)	Stress (81)

Ambiguous words are labelled in original dictionaries and shaded in grey. They are removed and replaced with the next most frequent non-ambiguous word.

Table S2: Proportion of field notes classified as sentiment negative/neutral/positive in “Strengths” and “Areas of Improvement” sections, from a modified AFINN/BING/NRC dictionary, according to “clinical encounter overall rating” strata: 1 (low) to 5 (high).

Strengths Text				
**Dictionary**	**Overall Rating**	**Negative**	**Neutral**	**Positive**
**AFINN**	**1**	0.095	0.444	0.460
	**2**	0.036	0.264	0.699
	**3**	0.031	0.230	0.739
	**4**	0.032	0.227	0.741
	**5**	0.033	0.184	0.782
**BING**	**1**	0.111	0.429	0.460
	**2**	0.043	0.223	0.734
	**3**	0.034	0.196	0.769
	**4**	0.031	0.185	0.784
	**5**	0.032	0.151	0.817
**NRC**	**1**	0.056	0.484	0.460
	**2**	0.027	0.255	0.718
	**3**	0.033	0.242	0.725
	**4**	0.028	0.236	0.736
	**5**	0.027	0.194	0.779
**Areas of Improvement Text**				
**AFINN**	**1**	0.238	0.373	0.389
	**2**	0.130	0.453	0.417
	**3**	0.084	0.558	0.358
	**4**	0.057	0.644	0.299
	**5**	0.029	0.707	0.264
**BING**	**1**	0.206	0.436	0.357
	**2**	0.122	0.455	0.422
	**3**	0.078	0.560	0.363
	**4**	0.049	0.648	0.303
	**5**	0.026	0.709	0.265
**NRC**	**1**	0.151	0.309	0.539
	**2**	0.066	0.384	0.551
	**3**	0.065	0.472	0.463
	**4**	0.047	0.591	0.363
	**5**	0.020	0.699	0.281
